# 

**Published:** 1893-04

**Authors:** 


### *EDITORIAL.*

The office of THE JOURNAL is in room 330 Equitable Building.

Address communications relating to the Editorial Department to DR. L. B. GRANDY, Atlanta, Ga., Box 431.

Business communications should be addressed to DR. M. B. HUTCHINS, Atlanta, Ga, Box 431.

All remittances should be made payable to "ATLANTA MEDICAL AND SURGICAL JOURNAL."

Articles for publication should reach this office not later than the 15th instant.

The editors are not responsible for views expressed by contributors.

### THE STATE MEDICAL ASSOCIATION.

The Association will meet at Americus on the 19th, 20th and 21st. There should be a large attendance. We understand that a good program is being arranged, and that every effort will be made to make this meeting successful in every way.

It is a well known fact that the Georgia Medical Association is not what it ought to be. The introduction of too much politics has destroyed in a great measure its usefulness, and injured its standing. So much more important is it then that the meetings should be attended by our most influential and representative men, in order that an organization may be perfected which will be an honor to our State profession. Georgia contains many medical men whose reputations are not confined to the limits of their State, and who could exert a strong influence for good in their State Association. But few of them attend the meetings. Why? Thereby hangs a tale—which we may tell at another time.

We understand that a movement will be inaugurated at this meeting looking to the correction of some of these evils. This will be to have County Associations organized, and to let these send their delegates to the State Association. We do not know the details of this proposed method, but believe it would work well. It is now in successful operation in other States. At any rate it would do much toward eliminating the element of politics.

There will be one matter to come before this meeting, doctor, which, of itself, will be of sufficient importance to bring you from home. That is the question of a State Board of Medical Examiners. It is now a gross neglect of duty, we think, for a State not to provide its citizens with the protection furnished by these boards. They stand between the people on one hand, and the unqualified practitioner and the dishonest quack on the other. This is a subject which concerns the people more than the doctors. But doctors are charitable, and those who compose this Association will be willing, we think, to give this matter their indorsement in the interest of the people. The chief interest which such a board can have for us doctors lies in the fact that it will give us a more capable and intelligent profession. The large number of incompetent young men that are sent out with diplomas every spring will be forbidden to practice, and the board would also keep out of our midst those vagrant and predatory quacks whom appropriate legislation is driving from other States.

We think we can say that this will be the most important matter to come before the Association. Be on hand, doctor, and give it your support.

